# A study to introduce National Early Warning Scores (NEWS) in care homes: Influence on decision‐making and referral processes

**DOI:** 10.1002/nop2.1091

**Published:** 2021-11-15

**Authors:** Philip Hodgson, Jane Greaves, Glenda Cook, Angela Fraser, Lesley Bainbridge

**Affiliations:** ^1^ Northumbria University Faculty of Health and Life Sciences Newcastle upon Tyne UK; ^2^ Gateshead Health NHS Foundation Trust Gateshead UK; ^3^ NHS Newcastle Gateshead CCG Gateshead UK

**Keywords:** care homes, early warning scores, frailty, older people, workforce development

## Abstract

**Aim:**

Early warning scores are commonly used in hospital settings, but little is known about their use in care homes. This study aimed to evaluate the impacts of National Early Warning Scores alongside other measures in this setting.

**Design:**

Convergent parallel design.

**Methods:**

Quantitative data from 276 care home residents from four care homes were used to analyse the relationship between National Early Warning Scores score, resident outcome and functional daily living (Barthel ADL (Barthel Index for Activities of Daily Living)) and Rockwood (frailty). Interviews with care home staff (*N* = 13) and care practitioners (*N* = 4) were used to provide qualitative data.

**Results:**

A statistically significant link between National Early Warning Scores (*p* = .000) and Barthel ADL (*p* = .013) score and hospital admissions was found, while links with Rockwood were insignificant (*p* = .551). Care home staff reported many benefits of National Early Warning Scores, including improved communication, improved decision‐making and role empowerment. Although useful, due to the complexity of the resident population's existing health conditions, National Early Warning Scores alone could not act as a diagnostic tool.

## 1 INTRODUCTION

Early warning scores (EWS) are tools designed to detect patients at risk of unexpected catastrophic deterioration in general, acute ward situations. Morgan et al. (1997) observed that many patients who were unexpectedly admitted to ICU had shown minor, premonitory disturbances of their physiology for several hours before action was taken. They suggested that early intervention on detection of these signs might improve patient outcomes and proposed a score system aggregated from ratings of vital signs that could be used to trigger intervention (Morgan et al., 1997). A wide variety of EWSs were subsequently developed (Downey, 2017, Gao, 2007). A rapid response system (RRS) comprises an EWS and a protocol for the urgent review of patients who reach a “trigger” level (Al‐Qahtani & Al‐Dorzi, 2010). It has proved difficult to demonstrate that any of these activities reduce morbidity and mortality in ways that can be attributed to the EWS protocol (Jones et al., 2016; Maharaj & Stelfox, 2016; Wendon et al., 2016). Despite this, the adoption of EWSs and RRSs is widespread in acute care and recommended in the UK by the NHS (NHS England, 2017), The National Institute for Health and Clinical Excellence (NICE, 2007), NCEPOD (NCEPOD, 2015) and professional organizations (ICS/FICM, 2013, RCP, 2012). It has also been officially endorsed in other national healthcare systems (ACSQHC, 2010; Berwick et al., 2006; CPSA, 2015; Institute for Healthcare Improvement, 2011). The Royal College of Physicians has led the development of a UK National Early Warning Score (NEWS; RCP, 2012; see Appendix S1) and its subsequent revision as NEWS2 (RCP, 2012) in order to encourage a standardized nationwide approach to EWS.

The ready availability of EWSs in acute care has led to them being used for other purposes than detecting patients who may be in the very early stages of deterioration. They are now used as severity of illness scores (RCP, 2012) and to prompt for the presence of early critical illness in situations where those caring directly for the patients do not have advanced clinical, diagnostic skills (Petersen et al., 2017).

Early warning scores is also being used in other clinical contexts, such as pre‐hospital and out‐of‐hospital care. There has been little research to support this repurposing of EWS. The Royal College of Physicians (RCP, 2012) has advocated that NEWS should be considered for implementation in prehospital assessment of acutely ill patients by “first responders,” e.g. the ambulance services, primary care and community hospitals, to improve the communication of acute‐illness severity to receiving hospitals (RCP, 2012 page xiii) and suggests that the full potential of NEWS for the assessment of the severity of acute illness in the community has not been reached (ibid page xiv). NHS England has adopted the use of NEWS2 in all secondary care and ambulance services since 2019, implementing NEWS2 as their common language for communicating concern regarding deteriorating patients (Benger, 2019).

This has stimulated interest in the use of NEWS in care home settings, with early findings indicating the potential for such scores to act as effective indicators of acute illness in comparison with routine observations (Barker et al., 2020). Care homes are home for many older people, and compassionate care requires that their necessary domestic and social care be not unnecessarily medicalized (Ward, 2018). Most of the carers in such facilities are unaccustomed to the assessment of clinical deterioration (Williams, 2016) and the use of NEWS might assist in such evaluation. However, the Royal College of General Practitioners (RCGP) has issued a position paper (Jan 2020) that does not endorse the routine use of NEWS2 in primary care (RCGP, 2020). Their reservations are three‐fold, firstly, NEWS2 has not been validated in primary care; secondly, they consider that there are difficulties with delivering routine NEWS2 at scale in primary care; and thirdly, its wide‐scale adoption would be costly. The RCGP recognizes the usefulness of NEWS2 in facilitating communication about patients between healthcare sectors and supports its judicious use whilst awaiting better evidence of validity in primary care (RCGP, 2020). The recognition of acute illness outside hospitals, and particularly in care homes has become an issue of international concern during the COVID‐19 pandemic. Comas‐Herrera et al. (2020) have reported that, in a survey of 22 countries, 41% of deaths from COVID‐19 occur in care homes. Systems based on NEWS2 scoring have been found to be an effective predictor of outcome in many countries including Italy (Paganelli et al., 2021), Brazil (Paixão‐Cortes et al., 2021) and Norway (Myrstad et al., 2020) as well as in the UK (Stocker et al., 2021).

## 2 DESIGN AND METHODS

This paper focuses on a study in which technology‐enabled NEWS was introduced into care homes in a locality in the north of England in 2017. The implementation featured the use of Bluetooth‐enabled clinical measurement (e.g. blood pressure, oxygen saturation, etc.) devices which are then synched with a tablet device to give an overall score, along with a short training programme provided by a practice educator to ensure appropriate use. The purpose of this implementation was to consider the factors affecting the adoption of NEWS in care homes and explore if NEWS alone was an indicator of illness in this population. Therefore, this study aimed to:

- Assess the relationship between NEWS score and frailty, cognitive impairment, dependency, functional ability and treatment outcomes in the acutely ill older care home resident;
- Explore care home staff, NHS community nurses working in care homes and GPs' views, experiences and barriers to the use of NEWS
- Examine the impact of the introduction of NEWS on clinical decision‐making processes in relation to treatment of the acutely ill older care home resident.

### 2.1 Design

The study used a two‐strand convergent parallel design featuring mixed methods (Cresswell & Plano Clark, 2012). This approach is characterized by collection and analysis of both quantitative and qualitative data with equal weight being given to the results.

The design included analysis of NEWS scores to assess the relationship between this score and other demographic and clinical data (strand one) and investigation of professionals' experiences of views of NEWS within the context of care homes (strand two). Data were collected between April 2017 and January 2018.

### 2.2 Data collection and process

#### 2.2.1 Strand one

All residents within the participating homes undertook baseline measures of NEWS (ranging from 1 = low risk to 10 = high risk), assessment of daily living using Barthel ADL index (Mahoney & Barthel, 1965; ranging from 0 = complete dependence to 100 = complete independence) and frailty using Rockwood clinical frailty score (Rockwood et al., 2005; ranging from 1 = very fit to 9 = terminally ill). All data were collected by the care home staff and practice educator as part of the home's routine care.

Data were collected as a baseline for all residents (*N* = 276), and then 356 additional measurements were collected for all scales for 158 residents across all four homes over an 8‐month period (ranging from 1 additional score to 12 additional scores) following triggers such as falls, vomiting or residents being generally unwell. Data were anonymized by the practice educator involved in the implementation of NEWS in this setting before being passed to the research team in a Microsoft Excel file.

#### 2.2.2 Strand two

When care home managers agreed to participate in the NEWS implementation project they and their team were also invited to take part in the qualitative arm of the evaluation study. Six weeks following the implementation of training for the use of NEWS by a practice educator in the home, staff were invited to participate in either a group or individual interview. A pragmatic approach was adopted for data collection which was reliant on the availability of professionals. The following topics were developed with the project steering group to be explored during the interview: influence of NEWS score on decisions to admit to hospital; other assessments taken into consideration (e.g. frailty, severity of the acute illness, functional status); practical enablers and barriers to carrying out a NEWS score with care home residents. Participants were also asked about situations where the NEWS score influenced decisions about continuing to care for the older person in the care home.

All interviews with care home staff were conducted one‐to‐one in a break room of the homes by the lead researcher. Interviews lasted for an average duration of 14 min. Healthcare professional interviews were conducted via telephone by the lead researcher, and lasted an average duration of 21 min.

### 2.3 Data analysis

#### 2.3.1 Strand one

Complete data were entered into SPSS Version 23 (IBM Corp, 2013) for analysis. Due to the potential variation in the data, descriptive analysis was initially undertaken to identify any trends between variables. Following this, to correlate outcomes, chi‐square calculations were used to test for trends and Cramer's V was used to test their significance.

#### 2.3.2 Strand two

Data were transcribed verbatim by the lead researcher in preparation for analysis. Individual interviews then underwent a process of thematic analysis (Braun & Clarke, 2006), commencing with the data being read, line‐by‐line, by multiple members of the research team to ensure quality and identify sections of data which highlighted initial codes and then themes, which could then be compared across the entire data set. Transcripts were entered into NVivo data analysis software (QSR International, 1999), to support and facilitate qualitative analysis by collating data highlighted under themes, as nodes.

### 2.4 Participants

A total of four care homes in the region took part in the investigation of NEWS, and all agreed to participate in the evaluation. A total of 276 residents living in care homes (one residential providing supervised care only, two providing residential and nursing care and one providing residential and dementia care) received a baseline measure (16 residents received incomplete NEWS scores where only partial measures were collected).

Strand two: although no staff refused to take part in the study, data were only collected in two homes (one nursing/residential and one residential dementia) as delays to implementation meant that the remaining homes had not been in receipt of NEWS for long enough to make a considered contribution. A total of 13 care home staff took part in interviews. A breakdown of participating homes and the roles of participants is shown in Table 1.

**TABLE 1:** Participant breakdown

Role	Number	Gender	Time in current home (range)	Experience in care home (range)
Manager	1	1 = F	18 months	28 years
Deputy Manager	1	1 = F	2 years	28 years
Nurse	1	1 = F	3 years	17 years
Senior Care assistant	5	1 = M 4 = F	5 months–7 years	2–20 years
Care assistant	5	1 = M 4 = F	3 months–6 years	5–24 years

Alongside this, four healthcare professionals (GP, older person nurse specialists, 24/7 rapid response nursing member and practice educator) working in the two participating homes agreed to take part in individual interviews. Topics discussed included:

- Experiences of, and barriers to, the use of NEWS in care home services
- Service and organizational impacts of NEWS in care homes
- Compatibility of NEWS with other organization procedures, systems and policies.

All participants were given information sheets and completed informed consent procedures before their interview. In order to maximize participant anonymity, contributions from this stage of the project will be assigned as healthcare partner, rather than individual role identifier codes.

## 3 RESULTS

A full breakdown of resident participant background can be found in Table 2.

**TABLE 2:** Resident participant breakdown

(a)	Resident participant breakdown
Female	171 (62%)
Male	105 (38%)
Mean age	84.65 years (56–100)
Residential care	127 (46%)
Nursing care	83 (30%)
Residential/dementia care	66 (24%)

### 3.1 Strand one

Results indicated at baseline that NEWS scores were found to be in the low‐risk range (1–4) with a mean of 1.15 (*N* = 260, *SD* = 1.45). As expected, mean Barthel ADL scores were in the severe dependency range (20–60) at 41.34 (*N* = 276, *SD* = 27.56) among the group. However, frailty scores were found to be between moderately (6) and severely frail (7) at a mean of 6.44 (*N* = 276, *SD* = 0.818).

### 3.2 Referral trends

Where subsequent measures were triggered, almost two‐thirds (66.2% *N* = 233) were referred to another service following observations being collected. The majority of these were to the resident's own GP (73.4% *N* = 174). Many resulted in a home visit (81.0% *N* = 192). A total of 31 (9.91%) measures taken for 26 residents resulted in hospital admission. Twenty two residents were admitted to hospital once, three residents were admitted twice and one resident was admitted three times.

### 3.3 Association between measures and outcomes

Spearman rank‐order correlation coefficient was used to identify associations between NEWS, Barthel ADL and Rockwood scores at all time points. This revealed a statistically significant relationship between Barthel ADL and Rockwood scores (*r* = −.757, *p* < .0001). However, this was not found to be the case between NEWS and Barthel ADL (*r* = −.033, *p* = .538) and NEWS and Rockwood (*r* = .063, *p* = .238).

Changes from baseline for the 356 additional triggers across all measures were mapped against whether a resident was referred, with what outcome and whether this resulted in a hospital admission. This revealed that greater escalation in each score resulted in an escalation of outcome, with higher levels of NEWS resulting in referral to another service. One variation was that higher NEWS and Rockwood scores resulted in the use of telephone advice, whereas higher changes in Barthel ADL scores resulted in home visits, most often from GPs.

Similarly, it was also found that there was a statistically significant link between NEWS score and hospital admissions (chi‐square = 0.573, *p* < .0001, Cramer's V = 0.405). However, while Barthel ADL was also found to be significant (chi‐square = 0.461, *p* = .013, Cramer's V = 0.326), links between Rockwood and hospital admissions were insignificant (chi‐square = 0.221, *p* = .551, Cramer's V = 0.156).

### 3.4 Strand two

Qualitative interviews undertaken with staff in two participating care homes, along with other health professionals, highlighted five key themes. These were (a) NEWS measurements as “proof,” (b) NEWS as efficient diagnosis, (c) NEWS as empowering communication, (d) NEWS as empowering role and (e) NEWS’ influence on decision‐making.

### 3.5 NEWS measurements as “proof”

Participants outlined that, prior to the implementation of NEWS, judgements on acute symptoms that residents presented with were based on subjective observations. One of the key benefits of NEWS, therefore, was the reassurance staff gained from basing their decisions on clinical measurements.

it's just the reassurance that you've got this proof. It's not pen and paper – people can't say you've made it up. (Carer)

NEWS measurements were valued as key sources of information that provided objective quantification of a resident's status:

Like, ill health, I’ll do the NEWS and say it comes back as a three, but usually their standard is a one, I know something is not right. Do it again in an hour's time – and if it's still getting higher… You've got proof there they aren't well. (Carer)

Thus, the ability to use NEWS to access quantifiable and measurable information about the resident was seen as a key benefit by participants.

### 3.6 NEWS as efficient diagnosis

Another reason NEWS was valued by the participants was the perceived benefits it had in terms of resident care and diagnoses. The ability to provide care home staff and other professionals with detailed information early in the process was seen as a key factor to identifying concerns efficiently.

Like, whereas beforehand we would just keep observing – now this tells us that we're on the right path, get that help. (Carer)

This efficiency was also important to staff who viewed NEWS as an ongoing process. The technology both provided the crucial measurements, but also made them readily available for frequent comparisons

The more information that we've got about someone then the easier it is to assess how quickly they need to be seen. (Healthcare partner)

Therefore, the ease of access to multiple, ongoing points of data was also seen as a key benefit to staff.

### 3.7 NEWS as empowering communication

Another core impact of NEWS in the care home setting was its impact on communication. This was viewed as providing better care and bridging a perceived gap in communication between care home staff and other health professionals.

Because we're using it, the paramedics use it, and the GPs use it, you're all on the same hymn sheet. (Carer)

Another consequence of this change in communication was the additional benefit of empowering care home staff. The inability to communicate with healthcare professionals reinforced the negative view staff previously had of their own role.

Obviously being a senior carer, we're still just care staff. Just with a senior role to it. So we've got no medical background. But now they [GPs] know that we're doing this… (Carer)

This improved communication and shared language was not just seen as a benefit when working with external partners, but it was also thought to improve internal communication, such as during handovers.

### 3.8 NEWS as empowering role

Participants reported feeling empowered by simply being involved in the NEWS processes and assessments. NEWS allowed staff to participate in the delivery of a “professional” service and make clinical judgements. This contrasted sharply with their pre‐NEWS negative views about their role.

It has reassured me, but it has boosted my confidence […] when people are saying, well, have you just checked this, and I can't say, “Well, actually, no I haven't.” Whereas now I can say I have. (Carer)

This sense of empowerment was viewed as particularly important when considering the contact care home staff have with other healthcare providers such as GPs, paramedics and the rapid response nursing team. With NEWS in place, staff suggested that they now felt more confident and empowered, and that this was reflected in the greater responsibility given to them by other healthcare services.

The doctor, we're giving them that. So it's saving them from doing that, because they're confident in us, that we've done it properly. (Carer)

This increased confidence was also perceived to have benefits for the residents in that it prompted improvements in care and allowed staff to be more engaged with referral processes.

You feel a bit more confident because obviously you've done the training and … I mean, nobody is perfect, but as you say the observation and that, you can make that decision there and then if… What needs to be done. (Carer)

### 3.9 NEWS and decision‐making processes

While NEWS was seen as having multiple positive impacts in terms of empowerment, communication and efficiency, it was still crucial to note that participants did not feel that it unduly influenced decision‐making. Despite speaking positively about the processes, participants still said their main source of information was “knowing the person,” rather than NEWS.

And I think that's where it's given them the confidence. It's the fact that that score changes. If it doesn't, they also know that I’m not bothered. If you've got… If you… We know our residents – if you're going with your gut, you go with your gut. That doesn't change, you know. (Home manager)

### 3.10 Barriers to the use of NEWS

Although participants were asked to identify barriers to the use of NEWS, no negative comments on the nature of the tool were discussed. Only issues relating to the reliability of the technology in the home were raised, although these were overcome by the use of paper‐based recording.

## 4 DISCUSSION

Although the positive findings on NEWS as a predictor of outcomes in this paper echo with much of the international literature on this subject (Paganelli et al., 2021; Paixão‐Cortes et al., 2021; Stocker et al., 2021), the introduction of EWSs to care homes requires an understanding of the problems they can be used to address and what their adverse consequences might be. The clinical context of a care home is very different to that of an acute hospital ward, where EWS systems are usually employed. Care home residents live with existing complex multi‐morbidities and high levels of frailty. Furthermore, many people die in care homes and recent figures for England showed that 36.7% of deaths in people aged 85 and over occur in care homes (Public Health England, 2019). As such, the specific implications of using EWS in this context require further consideration.

### 4.1 The relationship between NEWS, frailty and daily function in resident outcomes

The Royal College of Physicians' (RCP, 2012) national working party was unconvinced that it was necessary to apply a weighting to the NEWS aggregate score on the basis of age (RCP, 2012, p 11) despite evidence that its use improved the function of EWSs (Smith, 2008, Subbe, 2006, Subbe et al., 2007). Other vital signs and physiological status not included in NEWS such as frailty and mobility (Brabrand & Kellett, 2014) may be significant indicators of deterioration in the care home setting.

Although existing work on NEWS in care home settings has suggested comparability between NEWS scores in care homes and the general out‐of‐hospital setting (Barker et al., 2020), this study has illustrated potential key factors in terms of both daily function and frailty. Firstly, it is unsurprising that the care home population studied here displayed high level of frailty at the outset, and this provides a strong and influential context for the care home staff's decision‐making. Secondly, although the NEWS score itself was shown to have an impact on resident outcomes, functional daily living scores were also seen to be significant in influencing both the type and amount of care given. As such, further exploration of which measure is the most appropriate indicator of decline, and greatest influence on the care home staff's knowledge of the client, may be required.

These debates about relevance of physiological parameters in the context of use of NEWS with the frail, older care home population should be carefully considered when their use in this setting is being considered. Other issues, such as frailty, reduced mobility, confusion, delirium and cognitive impairment have been reported to affect mortality rates (Evans, 2014), the outcome of surgery (Lin, 2016), the results of intensive rehabilitation programmes (Singh, 2012), and how acute illness present (Craswell, 2016) should all be considered alongside the use of NEWS in care homes.

### 4.2 Experiences and barriers to the use of NEWS

Evidence on the use of NEWS in care homes to date is limited, both in the UK context and beyond. Unsworth and Bell (2017) evaluated the experience of adopting a Digital Care Home tablet in one locality. Three assessments were incorporated in this tablet: NEWS, the Malnutrition Universal Screening Tool (MUST) and ABBEY pain score. Pre‐ and post‐comparisons of a range of variables, including GP visits, referral to NHS services and 999 calls, indicated that there was a reduction in GP visits, emergency ambulance usage and accident and emergency attendance, yet there was an increase in recover‐at‐home contacts. Care home staff also suggested that NEWS readings helped improve communication with other healthcare staff. Similar outcomes were reported by West Hampshire CCG (2017) in relation to how NEWS in care homes is supporting a clear process for escalation of care as well as improving communication healthcare professionals (Patel, 2018).

Evidence from this study demonstrates that although NEWS has some impact on resident outcome, it is not being used as an early warning score for the earlier detection of incipient critical illness, but instead to assist care home staff to convert their detailed and nuanced understanding of residents' needs and understanding into clear decision‐making processes and language that is acceptable to clinicians.

### 4.3 NEWS and decision‐making

Interview data suggest that care home staff valued and used NEWS as a decision‐making tool and communication aid. It provided the staff with a language for discussing deterioration that is increasingly common to healthcare partners with whom they need to discuss possible problems. Secondly, it provided confirmation and reassurance that the clinical suspicion of a problem that had led them to measure and calculate a NEWS score was a reality, particularly after a trigger event. The interviews revealed that staff were not previously confident in communicating their knowledge of residents who no longer “seemed right” in their opinion, and NEWS allowed them to do this. This can be crucial in both ensuring correct care decisions are made by the care staff, and protecting other services from undue burden. In a population containing complex health concerns, reliance on NEWS unmediated from individual knowledge could lead to increased referrals. Meanwhile, the role empowerment of care home staff and improved communication illustrated here are also potentially important outcomes independent of the NEWS score itself.

## 5 CONCLUSION

The implementation of technology‐enabled NEWS in the care home context has been shown to have many benefits in the care provided, particularly in the ability to highlight the need for hospital admission and the improvement communication and empowerment of staff. However, both its appropriate use by staff and its ability to capture potentially vital related issues, such as frailty and function, requires further attention and ongoing research.

## 6 STRENGTHS AND LIMITATIONS

This paper uses a mixed‐methods design to help illustrate and explore the key outcomes for the introduction of EWS approaches in the care home setting. However, it does include several limitations. Firstly, due to logistical issues delaying the roll out of the tool in a greater number of homes, further large‐scale research is required to identify generalizable trends. Similarly, as data were collected during a limited timeframe, further longitudinal research is required. It is also noticeable that participant qualitative feedback from care staff was universally positive. The reasons for this are unclear, but could represent staff gratitude for involvement in the programme or the research team being associated with its roll out. Such issues require consideration and further research to follow up.

## AUTHOR CONTRIBUTIONS

Philip Hodgson: study development, data collection and analysis, write up; Dr Jane Greaves: data analysis and write up; Glenda Cook: study development, data collection and analysis, write up; Angela Fraser: data analysis and write up); Lesley Bainbridge: data analysis and write up.

## ETHICAL APPROVAL

Research ethics approval to undertake the study was obtained from the Faculty of Health and Life Sciences, Northumbria University on 6th December 2016.

## Supporting information

Appendix S1

## Data Availability

The data that support the findings of this study are available on request from the corresponding author. The data are not publicly available due to privacy or ethical restrictions.
