# Supplementary material for: A study to introduce National Early Warning Scores (NEWS) in care homes: Influence on decision‐making and referral processes
Source: Nurs Open. 2021 Nov 15;9(1):519–26. doi: 10.1002/nop2.1091 (PMC8685833; doi:10.1002/nop2.1091)
Supplement: Supplementary file 1 — Appendix S1 [file NOP2-9-519-s001.docx]

Supplement one: Overview of NEWS

Available at: [www.nice.org.uk/advice/mib205/chapter/The-technology](http://www.nice.org.uk/advice/mib205/chapter/The-technology)

The [National Early Warning Score](https://www.rcplondon.ac.uk/projects/outputs/national-early-warning-score-news-2) (NEWS2) is a system for scoring the physiological measurements that are routinely recorded at the patient's bedside. Its purpose is to identify acutely ill patients, including those with sepsis, in hospitals in England. The NEWS2 scoring system measures 6 physiological parameters:

- respiration rate
- oxygen saturation
- systolic blood pressure
- pulse rate
- level of consciousness or new-onset confusion
- temperature.

A score of 0, 1, 2 or 3 is allocated to each parameter. A higher score means the parameter is further from the normal range. Appropriate clinical responses are given for threshold (trigger) levels, with a recommendation to review and agree these locally:

- Low risk (aggregate score 1 to 4) – prompt assessment by ward nurse to decide on change to frequency of monitoring or escalation of clinical care.
- Low to medium risk (score of 3 in any single parameter) – urgent review by ward-based doctor to determine cause and to decide on change to frequency of monitoring or escalation of clinical care.
- Medium risk (aggregate score 5 to 6) – urgent review by ward-based doctor or acute team nurse to decide on escalation to critical care team.
- High risk (aggregate score of 7 or over) – emergency assessment by critical care team, usually leading to patient transfer to higher-dependency care area.
